# Turbid but accurate: automating lysostaphin quantification including uncertainty quantification

**DOI:** 10.1186/s12934-025-02911-w

**Published:** 2026-01-10

**Authors:** Lisa Prigolovkin, Michael Osthege, Maximilian Siska, Josefin Sander, Anja Hoffzimmer, Wolfgang Wiechert, Christian K. Desiderato, Christian U. Riedel, Marco Oldiges

**Affiliations:** 1 Institute of Bio- and Geosciences, IBG-1: Biotechnology, Forschungszentrum Jülich GmbH, Wilhelm-Johnen-Straße, 52428 Jülich, Germany; 2 Institute of Biotechnology, RWTH Aachen University, Worringer Weg, 52074 Aachen, Germany; 3 Computational Systems Biotechnology (AVT.CSB), RWTH Aachen University, 52074 Aachen, Germany; 4 Microbial Biotechnology, Biology Department, Ulm University, Albert-Einstein-Allee 11, 89069 Ulm, Germany

## Abstract

Conventional methods for measuring antibacterial activity, such as disk-diffusion assays, have limitations in quantitative reliability and require long incubation times making them unsuitable for high-throughput applications. To address these limitations, we automated a turbidity-based assay using readily available equipment and Bayesian data analysis, enabling accurate and precise antibacterial quantification from high-throughput experiments. In this study, we demonstrate the method applied to lysostaphin, a potent anti-staphylococcal agent and promising candidate for therapeutic applications. The turbidity assay monitors optical density changes upon lysostaphin-induced lysis of a susceptible *Staphylococcus* strain. We validated the use of autoclaved *Staphylococcus carnosus* TM300 as suitable indicator strain and optimized assay conditions for dynamic range of 0.63–10 mg L^−1^ lysostaphin. Our integrated approach provides a robust, scalable, and reproducible platform for quantifying active lysostaphin, paving the way for its application in high-throughput screening and process development. We believe that the approach is adaptable to other turbidity-based assays, such as those assessing endolysin activity.

## Introduction

Lysostaphin, a 27 kDa endopeptidase first isolated over 60 years ago from *Staphylococcus simulans* biovar *staphylolyticus* [1], remains one of the most potent and specific antibacterial compounds against staphylococci harboring pentaglycine cross bridges in their cell wall [2]. Lysostaphin consists of two domains, one catalytic and one cell-wall-targeting domain, connected by a flexible linker [3]. The C-terminal cell-wall-targeting domain directs the binding of the enzyme to the staphylococcal peptidoglycan layer thereby coordinating the N-terminal catalytic domain to its substrate [4]. The catalytic domain cleaves the pentaglycine cross bridges in the cell wall between the third and the fourth glycine residue [5]. Treatment with lysostaphin causes nanoscale perforation in the peptidoglycan layer, degradation of the cell wall with subsequent cell swelling and rapid lysis of the targeted staphylococcal cells [6–8].

With lysostaphin being highly effective against antibiotic resistant *Staphylococcus aureus* strains, including methicillin- and vancomycin-resistant strains [9, 10], its recombinant production and therapeutic potential continue to be actively explored [11]. A prerequisite for further development is the availability of robust and scalable methods to quantify its enzymatic activity. However, the quantitative assessment in the field of bacteriocins remains a major bottleneck. Conventional analytical methods such as ELISA or HPLC are rarely applicable, as bacteriocins are typically produced in complex culture supernatants, where they occur at low concentration and are masked by substantial background interference [12]. Consequently, activity based assays such as disk diffusion and minimum inhibitory concentration (MIC) assays remain widely used and have been applied for the quantification of lysostaphin [10, 13]. While disk diffusion assays are a simple method and provide a straightforward readout of bacteriolytic activity, they lack quantitative reliability due to diffusion-dependent variability and non-linear relationship between inhibition zone size and concentration. Moreover, relatively high concentrations of more than 50 mg L^−1^ are required for susceptibility testing of strains [10]. In contrast, the MIC assay allows for a comparatively straightforward quantification of the inhibitory concentration, but its quantitative resolution is constrained by the use of twofold serial dilutions. As a result, minor changes in activity cannot be resolved, and transitions between dilution steps may result in apparent twofold differences in MIC values that do not accurately reflect the actual magnitude of the effect. Its long incubation times of up to 24 h further hinder high-throughput applications [10]. Photometric assays are compatible with liquid handling systems and preferable for quantifying lysostaphin activity in a high-throughput context [14]. In literature, various approaches are reported including ninhydrin-based colorimetric assays, fluorometric FRET-based assays, and one dye release assay [15–17].

A commonly used method for assessing lysostaphin activity is the turbidity assay, which monitors the lysis of a susceptible bacterial indicator strain [1, 10, 18–20]. The progressive lysis of the indicator strain changes the optical cell properties and reduces light scattering, usually recorded as a decrease in absorbance over time, i.e., reduction of turbidity. *Staphylococcus aureus* strains are frequently used for assessing lysostaphin-mediated lysis in turbidity assays due to their clinical relevance [21]. However, biosafety concerns and compatibility with safety level S1 laboratory operation may necessitate alternative, non-pathogenic surrogate strains in routine workflows. The food-grade, non-pathogenic *Staphylococcus carnosus* TM300 features the characteristic pentaglycine cross bridges in its peptidoglycan layer and can thus serve as a suitable alternative indicator strain [22, 23].

Despite its advantages, the turbidity assay presents practical challenges. The preparation of freshly grown indicator strain for each turbidity assay measurement requires scheduling at least one day in advance and can hinder standardization between experiments due to variable factors such as harvest time or growth in complex media. Manual pipetting steps including standard dilution generation and sample pipetting are susceptible to human error and introduce operator-dependent variability. Given the rapid lysis reaction proceeding within a few minutes, minor deviations in the timing of indicator strain addition can result in inconsistencies. Moreover, interpreting the resulting kinetic data may involve subjective visual inspection of absorbance curves, which can further limit reproducibility.

Several studies have proposed quantification strategies based either on the turbidity reduction after a defined incubation time or on the rate of turbidity decrease over time [10, 24, 25]. However, the use of inconsistent metrics, such as the time or bacteriolytic units, hinders comparability across studies. Because the turbidity assay is compatible with the microtiter plate (MTP) format and readily available laboratory equipment, it can support high-throughput workflows [26]. These workflows generate large volumes of data in form of kinetic turbidity trajectories for individual MTP wells, underscoring the need for standardized, automated data-processing pipelines. Such tools are essential for ensuring consistent and objective result interpretation, particularly in large-scale screening workflows [27].

To overcome these challenges, we developed an automated version of the turbidity assay for quantifying lysostaphin activity in MTP format, enabling parallel analysis of eight samples across a dilution range from 1 to 256. We evaluated the use of autoclaved vs. non-autoclaved *S.* *carnosus* TM300 as indicator strain for reduced manual workload and investigated the influence of the used biomass concentration on the assay readout. Additionally, we introduce a Bayesian process model that quantifies active lysostaphin concentration of all samples from a kinetic description of the exponentially decaying turbidity across all assay wells simultaneously. The automation of both the assay and subsequent data analysis greatly enhances reproducibility, comparability, and throughput of the turbidity assay.

## Methods

### Strain maintenance and cultivation parameters

*S. carnosus* TM300 [23] served as cellular substrate in the turbidity assay and was cultivated in LB or BHI medium. All cultivations were carried out in shake flasks with a 1/10 filling volume at 37 °C and 250 rpm on a rotary shaker with 25 mm shaking diameter. For cryo conservation, 50 mL LB were inoculated from a single colony and incubated overnight. Cells were centrifuged (4000 × *g*, 20 °C, 5 min) and concentrated in 0.9% (w/v) NaCl solution to an optical density (OD) of 20 at 600 nm. OD_600 nm_ was measured using a UV-1800 spectrophotometer (Shimadzu, Kyoto, Japan) within a linear range of 0.1–0.3. The cell suspension was mixed 1:1 using 50% (w/v) glycerol and stored in aliquots at − 80 °C.

### Indicator strain preparation

100 µL *S. carnosus* TM300 cryo culture were used to inoculate 50 mL BHI medium and incubated overnight. For preparation of viable indicator strain suspensions, cells were harvested by centrifugation (3200 × *g*, 12 min) and washed in 1X phosphate buffered saline (PBS, pH 7.4) before they were resuspended to the desired OD_600_ _nm_. For autoclaved indicator strain stocks, the cell suspension was heat-inactivated at 121 °C and 2 bar for 30 min. Cells were harvested by centrifugation (3200 × *g*, 12 min) and washed in PBS. Resuspended cells were transferred to 2 mL reaction tubes and centrifuged at 3200 × *g*, for 12 min. The supernatant was discarded and cell pellets were stored at − 20 °C for up to a week or at − 80 °C for up to 8 months until further use in turbidity assay. Cell morphology was checked using a Zeiss Axio Imager 2 (Carl Zeiss, Oberkochen, Germany).

### Turbidity assay

An aliquot of *S. carnosus* TM300 was thawed and resuspended in PBS (pH 7.4) to the desired OD_600 nm_. Commercially available lysostaphin from *Staphylococcus simulans* (Sigma-Aldrich, St. Louis, USA) with specific activity ≥ 3000 unit mg^−1^ was dissolved in PBS (pH 7.4), sterile filtered and stored at − 20 °C until use.

For the manual turbidity assay, 200 µL sample was added to a well and serially diluted twofold in PBS (pH 7.4) to a well volume of 100 µL. The assay was initiated by adding 100 µL *S. carnosus* TM300 suspension to each well using a multichannel pipette, yielding a reaction volume of 200 µL. The assay plate was immediately transferred to a microplate reader to measure absorbance at 600 nm in kinetic cycles for 1 h at room temperature, with a 5 s of linear shaking (1 mm amplitude) between cycles to prevent cell sedimentation.

The automated turbidity assay was performed on the Opentrons OT-2 system equipped with a 1000 µL Gen2 single channel pipette, 300 µL Gen2 multichannel pipette and a temperature module (Opentrons Labworks Inc, Long Island City, New York, USA). The samples were cooled to 10 °C in a 96-well flat bottom MTP on the temperature module. The indicator strain suspension and PBS were provided in a 12-column trough. Lysostaphin standard was twofold diluted in PBS in 1.5 mL reaction tubes resulting in a final volume of 500 µL per concentration. 100 µL of each standard dilution was transferred in triplicates into the first three columns of the assay MTP. After mixing five times, 200 µL of each sample was transferred into the wells of column 4. The wells of columns 5–12 received 100 µL PBS. Samples were then serially diluted twofold by transferring 100 µL from the wells in column 4 to 5, and subsequently across the wells of the following columns. After each dilution step, solutions were mixed thoroughly by pipetting eight times up and down to ensure homogeneity. From each well of the last column, 100 µL were discarded for uniform filling levels of 100 µL per sample. The assay was initiated by adding 100 µL *S. carnosus* TM300 suspension to each well. The MTP was then manually transferred to a microplate reader (Infinite M Nano +, Tecan, Switzerland) for measurement of absorbance at 600 nm for 60 min. Unlike the manual protocol, an initial 20 s shaking interval (1 mm amplitude) was included. The robotic script for OT-2 operation is provided in the corresponding GitHub repository [28].

### Data analysis

Each turbidity assay run generates 96 time series of 600 nm absorbance, each recorded at 44 time points over the course of one hour. By the end of that hour, all turbidity trajectories have reached a lower limit.

These trajectories can be described as an exponential decay, starting at an initial value ${a}_{initial}$ corresponding to the absorbance of *S. carnosus* TM300 at the beginning of the assay. The half-time in the assay well ${t}_{1/2, well}$ [h] depends on the active lysostaphin concentration ${k}_{assay}$ [µM  _LSS_] and the specific decay half-time ${t}_{1/2}$ [h µM_LSS_^−1^]. The background absorbance ${a}_{background}$ resulting from indicator strain cell debris is accounted for as a lower-limit of the decay kinetic. With these three parameters the turbidity kinetic in one assay well can be described by the following function:

1 $$\begin{aligned} a(t) = a_{{initial}} - \left( {a_{{initial}} - a_{{background}} } \right) \cdot & \\  \left( {1 - exp\left( { - \frac{{{\mathrm{ln}}(2)}}{{t_{{1/2}} }} \cdot k_{{assay}} \cdot t} \right)} \right) \\ \end{aligned} $$

Measurement noise was modeled as normally distributed with a previously unknown standard deviation ${\sigma }_{turb}$ that is fitted together with the other model parameters. Some parameters are vectorised across multiple wells in the dataset and will be written using vector notation, e.g. ${k}_{assay}^{\to }$. The model was fitted with Bayesian inference, implemented using the probabilistic programming library PyMC [29] (version 5.16.2). Details are described in the corresponding results chapter.

## Results

### Autoclaved vs. viable *S. carnosus* TM300 indicator strain

The turbidity assay relies on monitoring the decrease in absorbance of a susceptible indicator strain suspension upon addition of lysostaphin-containing samples. Using viable indicator strain requires manual preparation prior to each turbidity assay run. These time-sensitive preparation steps add considerable complexity to the overall workflow. Since lysostaphin is both active against dividing and non-dividing cells [30], the utilization of the latter is an option. Accordingly, *S. carnosus* TM300 cells may be inactivated through autoclaving without compromising their susceptibility to lysostaphin [31]. The use of autoclaved cells may facilitate the long-term storage of indicator strain stocks, thereby improving the overall handling and flexibility of the turbidity assay, since the indicator strain can be prepared independently of the experimental timeline.

**Fig. 1:**
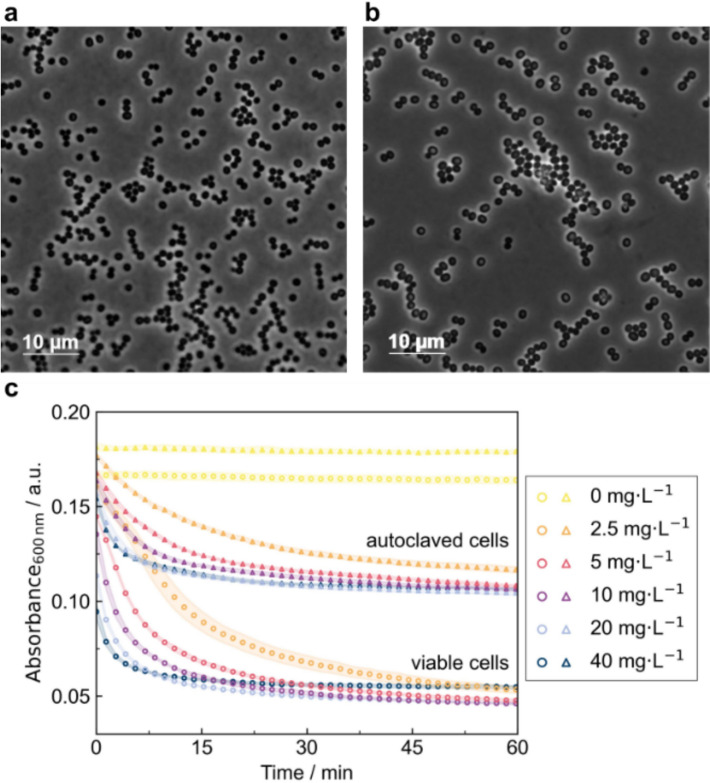
Microscopic images and comparison of autoclaved vs. viable *S. carnosus* TM300 cells in turbidity assay being both susceptible towards lysostaphin. **a** Autoclaved *S. carnosus* TM300 cells and **b **viable *S. carnosus* TM300 cells before turbidity assay were observed under 1:1000 magnification. Both cell populations exhibit intact morphology. **c **Absorbance at 600 nm of autoclaved (▲) and viable (●) *S. carnosus* TM300 suspension (OD_600 nm_ = 1) was measured upon exposure to lysostaphin standard (0–40 mg L^−1^) in kinetic cycles for 60 min using a microplate reader. Mean and standard deviations of n = 4 technical replicates are shown

Here, we tested both autoclaved and viable *S. carnosus* TM300 suspensions as substrate in turbidity assay. Microscopic analysis of *S. carnosus* TM300 cells revealed that both autoclaved (Fig. 1a) and viable cells (Fig. 1b) maintained intact morphology. No visible differences in cell shape or structural integrity were observed between the two conditions, indicating that autoclaving did not visibly disrupt the cellular structure. For turbidity assay, lysostaphin standard was prepared in twofold serial dilution in PBS (pH 7.4) in a concentration range of 2.5—40 mg L^−1^ while PBS served as negative control. Upon addition of the autoclaved (OD_600 nm_ = 0.98) and viable cell suspension (OD_600 nm_ = 1.00), the absorbance at 600 nm was monitored for 60 min (Fig. 1c). Both autoclaved and viable *S. carnosus* TM300 cells showed a clear absorbance reduction after the reaction start, proving susceptibility towards lysostaphin regardless of cell viability. The absorbance instantaneously decreased with a high initial rate, followed by a gradually decreasing rate until reaching the lower absorbance limit, indicating depletion of available pentaglycine substrate. The reaction speed increased with lysostaphin concentration from 2.5 mg L^−1^ to 40 mg L^−1^. For concentrations of 5 mg L^−1^ lysostaphin and higher, the reaction proceeded so quickly that the initial absorbance drop could not be captured, since it happened during the transfer of the MTP into the reader. This highlights the need for a fast and reproducible measurement start, as well as a robust quantification method able to deal with data from different runs.

The kinetic trajectories followed a similar behavior for both viable and autoclaved cells, showing high reproducibility among replicates, with a standard deviation in the range of ± 0.007 arbitrary (absorbance) units (a.u.). Strikingly, reactions with autoclaved indicator strain suspension did display higher initial (0.18 a.u.) and residual absorbance (0.11 a.u.) compared to the reactions with viable indicator strain (0.17 a.u. and 0.05 a.u.). Accordingly, the signal change of the autoclaved cells was only approximately 64% of that observed for the viable cells. Interestingly, this phenomenon has been reported for the lysis of *Staphylococcus aureus* cell suspension, where the turbidity reduction of autoclaved cells achieved only approximately 50% of that of the viable cell suspension [1]. Autoclaving of the cell suspension may have led to protein denaturation and aggregation [1, 32] and Maillard reactions between amino acids and sugars increasing the baseline absorbance.

The reduced absorbance range upon usage of autoclaved cells in contrast to viable cells can impact the analytical performance of the turbidity assay in two ways. First, the compressed signal changes provide less separation between lysostaphin concentrations, which can lower the accuracy of titer estimates. Second, the lower absorbance reduction makes the turbidity assay more susceptible to measurement noise, which may slightly reduce precision. Nevertheless, the use of autoclaved cells offers several advantages: Absorbance trajectories from autoclaved cells closely matched a textbook exponential decay trajectory. Autoclaving improves biosafety by eliminating cell viability, thereby removing the risk of contamination. This is especially advantageous in the use of pathogenic indicator strains like *Staphylococcus aureus*. Moreover, the metabolic inactivity of the cells prevents any unintended interactions or alterations in the assay environment. This also enables long-term storage of the cells, allowing the preparation of indicator strain batches for larger campaigns of turbidity assays. Storage of autoclaved *S. carnosus* TM300 cells was possible for at least 8 months at − 80 °C without affecting the kinetic trajectory in turbidity assays [28]. Using the same batch of cells enhances reproducibility and comparability across assays. In addition, handling and flexibility in preparation of the assay are greatly improved, as cultivation of the indicator strain before each assay is no longer necessary.

### Impact of indicator strain starting biomass concentration

**Fig. 2:**
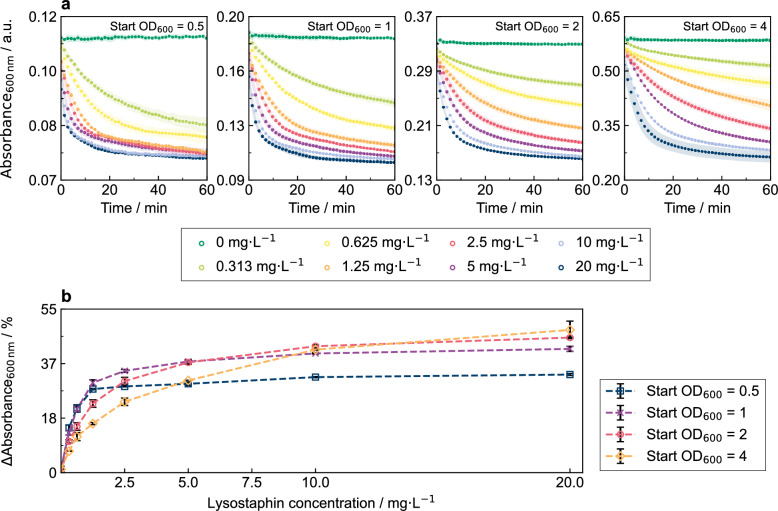
Comparison of different *S. carnosus* TM300 starting biomass concentrations in turbidity assay influencing the discernible active lysostaphin concentration range. **a **Absorbance at 600 nm of autoclaved, frozen *S. carnosus* TM300 suspensions of OD_600 nm_ of 0.5, 1, 2, and 4 in PBS was measured upon exposure to lysostaphin standard in kinetic cycles for 60 min using a microplate reader. Lysostaphin standard of the concentration 20 mg L^−1^ was supplemented with 0.5% (v/v) Tween 80 and diluted in PBS in twofold dilution series. **b **Turbidity reduction (∆Absorbance_600 nm_) of the *S. carnosus* TM300 suspensions for each tested OD_600 nm_ after 15 min in dependence of the lysostaphin standard concentration (0–20 mg L^−1^). Mean and standard deviations of n = 3 technical replicates are shown

To assess the effect of the starting biomass concentration on the lytic reaction, we conducted a turbidity assay testing a varying starting optical densities (OD_600_ _nm_ = 0.5, 1, 2, 4) of autoclaved *S.* *carnosus* TM300 (Fig. 2a). For each tested OD_600 nm_, exposure to lysostaphin led to an immediate absorbance reduction, which proceeds faster for higher lysostaphin concentrations. Higher starting biomass concentrations of the indicator strain increased the absorbance range accessible through the lysis reaction, which can be explained by the proportionally lower residual absorbance limit. For a starting optical density of 4, the residual absorbance corresponded to 60 ± 1%, whereas it was 79 ± 1% for OD_600 nm_ of 0.5. This was accompanied by more stable kinetic trajectories when OD_600 nm_ of 2 or 4 was used in contrast to OD_600 nm_ of 0.5 and 1.

In addition, the distinction between different lysostaphin concentrations became more pronounced at higher OD_600 nm_ of 2 and 4. This is also evident in the visualization of the relative absorbance reduction (ΔAbsorbance_600 nm_) after 15 min, plotted against the lysostaphin concentration (Fig. 2b). For OD_600 nm_ of 0.5, the dynamic range is limited to lysostaphin concentrations up to 1.25 mg L^−1^, as the absorbance reduction for lysostaphin concentrations ≥ 1.25 mg L^−1^ saturated at approximately 31 ± 2% ΔAbsorbance_600 nm_. For OD_600 nm_ of 1 and 2, the dynamic range extends to lysostaphin concentrations up to 10 mg L^−1^ with saturation at 41 ± 1% and 44 ± 2% ΔAbsorbance_600 nm_, respectively. For the even higher OD_600 nm_ of 4, the dynamic range was further extended to at least 20 mg L^−1^ with ΔAbsorbance_600 nm_ reaching a maximum of 48.0%. Thus, the measurable dynamic range increased with higher starting biomass concentration of the indicator strain. Since the lysostaphin effect on the indicator strain essentially corresponds to a single enzymatic reaction with the pentaglycine substrate, increasing the substrate concentration, i.e. OD_600 nm_ of indicator strain, allows higher substrate conversion and thereby expands the dynamic range of the assay. In the following experiments, an indicator strain suspension with OD_600 nm_ of 2 was used, representing a good compromise between the amount of indicator strain required and the measurable lysostaphin range. Samples with a lysostaphin concentration higher than 10 mg L^−1^ were diluted to fit within the dynamic range of the assay.

### Automation of turbidity assay

The turbidity assay involves several pipetting steps, including the preparation of standards, dilution of samples, and addition of the indicator strain. This becomes especially tedious when handling large sample sets. Manual pipetting also introduces variability, as the timing of the reaction initiation may differ between wells and assay plates, negatively affecting the reproducibility of assays. Automation of the assay can help to address these challenges by standardizing pipetting steps and increasing operator walk away time, thereby improving both efficiency and reproducibility.

The Opentrons OT-2 system, equipped with a 1000 µL Gen2 single channel pipette and 300 µL Gen2 multichannel pipette, enables automated pipetting of suspensions using disposable tips. The use of fresh tips is particularly favorable in the dilution series of standards to prevent carry over between wells. The assay incorporates the measurement of the lysostaphin standard in seven dilutions and one PBS sample as negative control in the first three columns of every assay plate ensuring a reliable readout. Eight samples, in this case 10 mg L^−1^ commercial lysostaphin, were automatically pipetted into the fourth column of the assay MTP and then serially diluted twofold in PBS across the subsequent columns up to the last column of the plate. After addition of the indicator strain suspension, the assay plate was immediately transferred to the plate reader by the operator for direct absorbance measurement at 600 nm.

**Fig. 3:**
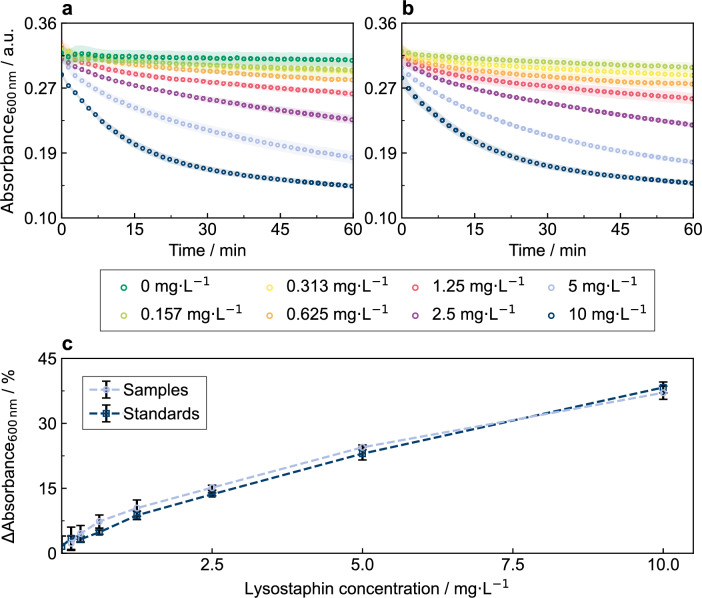
Kinetic trajectories in automated turbidity assay performed on the Opentrons OT-2 system. Absorbance at 600 nm of *S. carnosus* TM300 suspension (OD_600_ = 2) was measured upon exposure to lysostaphin standard (0–10 mg L^−1^). **a **Measurement of lysostaphin standard for wells in assay plate columns 1–3 that was diluted in reaction tubes on the Opentrons OT-2 deck. **b **Measurement of lysostaphin samples in dilution series directly prepared in the wells of columns 4–10 of the MTP showing high reproducibility and consistency with the independently diluted standards in columns 1–3. **c** Turbidity reduction (∆Absorbance_600 nm_) of *S. carnosus* TM300 suspension after 15 min for lysostaphin standards (■, assay plate columns 1–3) and lysostaphin samples (●, assay columns 4–10) in dependence of the lysostaphin standard concentration (0–10 mg L^.1^). Mean and standard deviation of n = 3–8 technical replicates are shown

The kinetic trajectories obtained from the automated turbidity assay (Fig. 3a,b) showed the same behavior and clear differentiation among lysostaphin concentrations as observed in the manual turbidity assay (Fig. 2a). The kinetic trajectories of the standard dilutions that were prepared in 1.5 mL reaction tubes (Fig. 3a) showed good agreement with the sample dilution prepared column-wise within the MTP (Fig. 3b). This is illustrated by the relative absorbance reduction (ΔAbsorbance_600 nm_) after 15 min, plotted against the applied lysostaphin concentrations (Fig. 3c). These results, together with the low standard deviation among eight technical replicates (Fig. 3b), demonstrate the high accuracy of the automated assay.

**Fig. 4:**
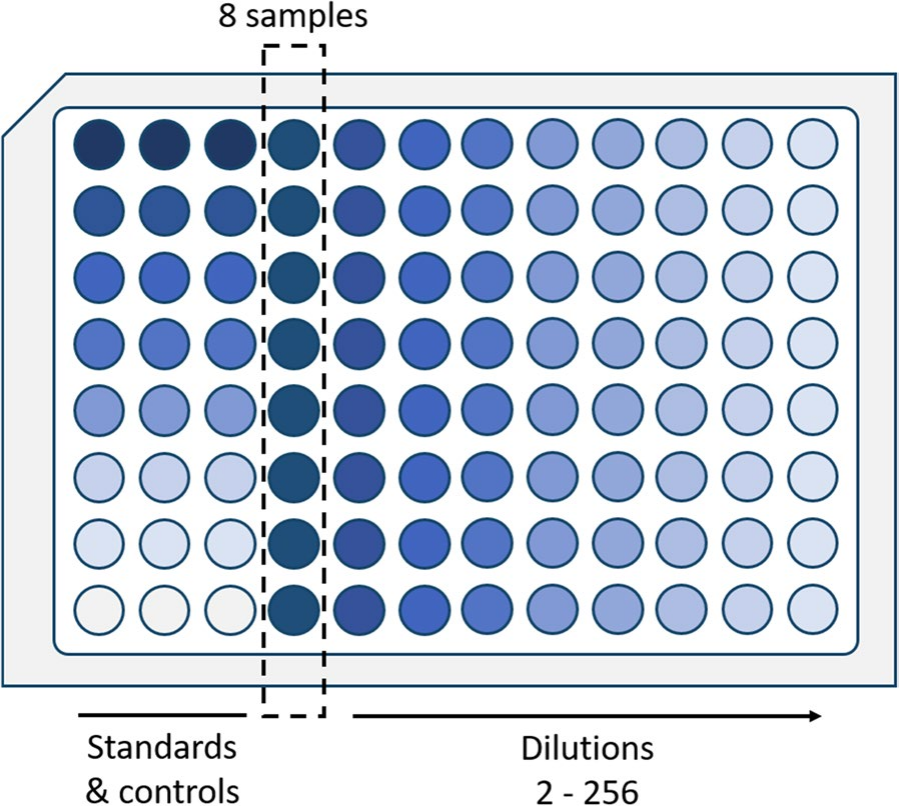
Turbidity assay plate layout for automated measurement of eight samples on the Opentrons OT-2 system. The first three columns contain the lysostaphin standard (0.156–10 mg L^−1^) and the negative control PBS. Eight samples are provided in column 4 and column-wise twofold diluted up to a dilution factor of 256. *S. carnosus* TM300 indicator strain suspension is then added column-wise to all wells of the MTP starting from the right site of the assay plate

The total assay duration for the measurement of eight samples using the presented automated assay layout (Fig. 4) was 1:27 h, comprising 27 min of pipetting, 1 h of plate reader measurement and negligible manual operator time for assay preparation. If necessary, the protocol can be adapted to measure a higher number of samples in less dilution steps or up to 72 undiluted samples on one assay MTP, further increasing the experimental throughput in large campaigns.

### Data analysis: model structure

Turbidity assays are analyzed using a range of different methods. Many quantification approaches rely on measuring the absorbance reduction at one point in time, e.g. 10 min after assay start, compared to a control suspension [24, 25]. Another approach is to determine the time required to reach half of the initial absorbance value [10]. These methods are prone to deviations in the assay start timing, as the reactions for samples and control suspension need to be started simultaneously for a reliable comparison. In addition, the assay start time itself is susceptible to operator-related variability. More accurate methods assess the rate of absorbance reduction per time in the linear range of the reaction [24]. However, they often rely on arbitrarily defined bacteriolytic units, which limits their broader applicability. While bacteriolytic units may allow for consistent data interpretation within a single study, they hinder meaningful comparisons across different studies. The lack of standardized quantification methods complicates the comparison of results across different studies and highlights the need for a more robust and widely applicable approach to active lysostaphin measurement.

To infer sample activities from measured absorbance kinetics, we developed a Bayesian model of the turbidity assay. In Bayesian inference, all uncertainty quantities, i.e., parameters, are treated as probability distributions rather than fixed values, allowing us to directly quantify uncertainty in activity estimates.

The model was implemented using the probabilistic programming library PyMC [29]. A hierarchical model structure was chosen to simultaneously account for well-to-well variability, arising from technical effects such as pipetting inaccuracy, while also combining information from multiple analytical replicates. This approach stabilizes individual estimates and provides credible uncertainty intervals.

**Fig. 5:**
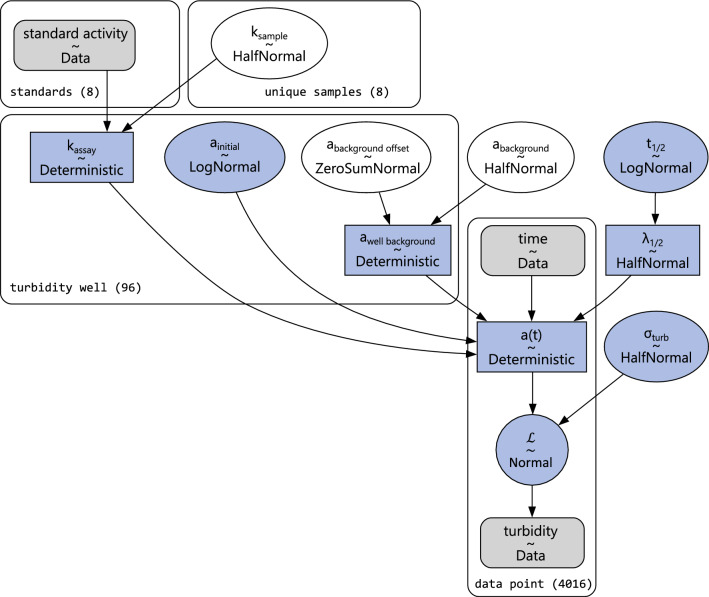
Plate model visualization of the turbidity assay model for eight unique samples measured in 72 turbidity assay wells and eight lysostaphin standards of known activity, measured in triplicates. The texts in the rounded boxes (e.g. “standards (8)”) indicate the name and size of the dimension that the vectorised model variables describe. Arrows show how upstream variables are used in the calculation of downstream quantities, eventually resulting in the predicted turbidity values a(t) that are compared to turbidity data by a normally distributed likelihood. The blue nodes indicate the variables involved in the model of one turbidity trajectory. Excluding the first 3 min, a total of 4016 observations remained to be described by the model

Inside the model, the turbidity trajectory is described by the function (1) introduced in Sect. "Data analysis", parametrized by half-time ${t}_{1/2}$, well-wise initial turbidity ${a}_{initial}$ and well-wise turbidity limit ${a}_{background}$. Model predictions of well-wise turbidity trajectories are connected to observed data assuming normally distributed observation noise with an unknown standard deviation ${\sigma }_{turb}$. Figure 5 shows the corresponding model variables marked in light blue.

A hierarchical prior was introduced to describe the turbidity background with a weakly informative $HalfNormal(\sigma =0.15)$ prior, while allowing each turbidity assay well to deviate from this group mean by a $ZeroSumNormal(\sigma =0.1)$ offset. The initial turbidity values are modeled independently for each well, with a $LogNormal(\mu =ln(0.35), \sigma =0.1)$ prior.

Ultimately, the sample activities of interest are those of the unique biological samples, irrespective of the number of technical replicates included in the turbidity assay. At the same time, the sample activities of the lysostaphin standards are known. Consequently, the model variable for well-wise activities ${k}_{assay}^{\to }$ is a vector of unknown sample activities, or fixed standard activities.

### Data analysis: inference results

The model was fitted by running 4 parallel chains of Markov chain Monte Carlo sampling using PyMC’s No-U-turn-Sampler (NUTS) for 2000 tuning iterations at a target acceptance rate of 0.9 and sampling 5000 draws from the posterior distribution. Convergence was checked by confirming Rhat < 1.01 for all variables using ArviZ [33].

**Fig. 6:**
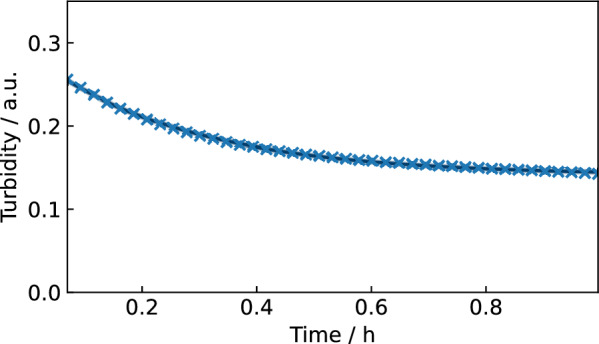
Exponential model for reaction kinetic in a MTP well with 10 mg L^−1^ lysostaphin. Fitted kinetic trajectory, data points as blue crosses (**×**) and a (very narrow) blue density band of the model fit in the background

Figure 6 shows the fitted kinetic trajectory of a turbidity assay reaction with 10 mg L^−1^ lysostaphin over the incubation time of 60 min. The first 3 min were excluded due to initial signal fluctuations, likely caused by inhomogeneous mixing at the start of the assay, which is later compensated by shaking in between the measurements.

Figure 6 shows that the model was not only capable of describing the turbidity kinetic observed from lysostaphin standards, but also fitted the previously unknown standard deviation of measurement noise at 0.0011 a.u. (with 94% HDI [0.00109, 0.00114] a.u.). Thus, standard deviation is almost two orders of magnitude smaller than the turbidity measurement values.

**Fig. 7:**
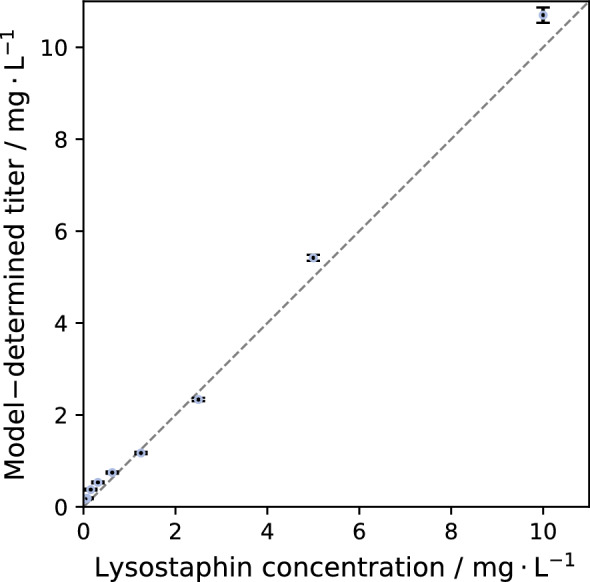
Model-determined lysostaphin titer in sample wells in comparison to the theoretically applied lysostaphin concentration measured in turbidity assay. Samples were measured in twofold dilution series ranging from 10 mg L^−1^ to 0.039 mg L^−1^. Mean and standard deviation of each concentration (0.16–10 mg L^−1^) assessed in n = 8 technical replicates are shown. Experimental data are taken from Fig. 3

To validate the model, it was applied to quantify lysostaphin concentration of the previously measured samples shown in Fig. 3b. The model analysis of the assay plate containing 72 sample wells finished in 14:10 min including the time taken for data import, model definition, and MCMC parameter estimation for uncertainty quantification of model parameters and sample titers. Given the runtime of less then 15 min for 96 samples, the approach can readily be applied to larger datasets without incurring prohibitive computation time. The lysostaphin titers estimated by the model correlated well with the known lysostaphin concentrations (Fig. 7).

Across most samples, the predicted titers varied only slightly from the actual values. The 10 mg L^−1^ and 5 mg L^−1^ samples were slightly overestimated (7.0% and 8.4%), whereas the 2.5 mg L^−1^ and 1.25 mg L^−1^ samples were slightly underestimated (− 6.6% and − 6.3%). At lower concentrations, the relative error increased because small absolute deviations translate into large relative error as the values approached zero. For the 0.63 mg L^−1^ sample, the relative error was 19.5%. For the 0.31 mg L^−1^ and 0.16 mg L^−1^ samples, the relative error increased to 70.2% and 139.2%, respectively. These results show that the model provides accurate titer estimates between 0.63 mg L^−1^ and 10 mg L^−1^. Below this threshold, the accuracy of the predictions decreases markedly. Accordingly, the effective sensitivity range of the turbidity assay can be defined as 0.63–10 mg L^−1^.

Overall, the Bayesian data analysis approach presented here provides a robust method for determining lysostaphin titers with high precision and accuracy in high-throughput settings. In contrast to approaches that rely on absorbance reduction over time or simple linear regression, the method is more complex and requires basic understanding of probabilistic modelling. It also demands additional computational effort. Nevertheless, the model remains computationally efficient and can be executed on a standard laptop without difficulty. One inherent trade-off of this approach is its sensitivity to previously unknown experimental effects; for example, substantial changes in the background turbidity may impede data analysis and necessitate adjustment of model parameters. However, this sensitivity can also be advantageous, as it helps to reveal underlying experimental deviations that might otherwise remain unnoticed.

Our modelling approach can, in principle, be generalized to a broader range of kinetic assays. The essential requirement is the formulation of a hierarchical model structure that links known standard titers and unknown sample titers to a kinetic parameter that changes in a concentration-dependent manner. In the present study, this relationship was defined through the half-time of the turbidity reduction in the assay well ${t}_{1/2, well}$ [h] depending on the active lysostaphin concentration ${k}_{assay}$ [µM_LSS_]. For other turbidity-based assay, such as endolysin assays [34], the overall model structure could be transferred with only minor modifications, as the assays exhibit comparable turbidity-reduction kinetics. Nevertheless, assay-specific characteristics, including initial turbidity levels and background turbidity, must be appropriately adjusted. Additional effect, such as matrix interferences, may further necessitate the introduction of extra variables or model extensions. Assays that rely on different kinetic behaviors may not be adequately described using the same functional form. In such cases, an alternative kinetic model with hierarchical structure and a different concentration-dependent kinetic parameter, e.g. the slope, would be required to ensure accurate linkage between sample titers and assay readouts.

## Conclusion

We demonstrated the successful automation of the turbidity assay in a standardized MTP format using an Opentrons OT-2 pipetting robot, followed by automated data analysis. *S. carnosus* TM300 was used as the indicator strain. Although the use of inactivated indicator strain slightly reduced the resolution of the assay compared to the use of viable indicator strain, it greatly simplified handling and is favorable in terms of biosafety considerations. Moreover, the ability to store inactivated indicator strain batches reduced manual workload and improved comparability across assays, as a large batch of indicator strain can be prepared in advance and used in multiple experiments. Increasing the biomass concentration of the indicator strain expanded the dynamic range of the assay. A starting biomass concentration of OD_600 nm_ = 2 provided high resolution for samples containing less than 10 mg L^−1^ lysostaphin. Considering the implemented sample dilution up to a factor of 256, the assay format theoretically supports quantification of 0.63 mg L^−1^ to 2560 mg L^−1^ active lysostaphin. The assay layout may be easily adapted to increase throughput, for example to measure 24 samples in 3 dilutions or 72 undiluted samples. The model-based data processing enabled standardized quantification of active lysostaphin titers with uncertainty quantification, eliminating subjective data interpretations and the use of arbitrary activity units. The presented turbidity assay module lays the foundation for future high-throughput quantification and modelling of lysostaphin in applications such as strain library screening or bioprocess optimization. Furthermore, the hierarchical structure of standards, unknown samples and random effects on kinetic parameters could be adapted for other (bio)chemical kinetic assays, such as, for example, other turbidity-based endolysin assays.

## Data Availability

The datasets supporting the conclusions of this article are available in the JuBiotech/Supplement-to-Prigolovkin-et-al.-2025 repository, [10.5281/zenodo.17036232.
